# First record of *Lithurguscornutus* (Hymenoptera: Apoidea: Megachilidae) from Poland

**DOI:** 10.3897/BDJ.9.e75997

**Published:** 2021-12-07

**Authors:** Mikołaj Borański, Waldemar Celary, Jacek Jachuła

**Affiliations:** 1 The National Institute of Horticultural Research, Skierniewice, Poland The National Institute of Horticultural Research Skierniewice Poland; 2 University of Jan Kochanowski, Kielce, Poland University of Jan Kochanowski Kielce Poland

**Keywords:** solitary bee, pollinator, distribution, range, Central Europe

## Abstract

The paper presents the first record of *Lithurguscornutus* (Fabricius, 1787) in Poland. Until recently, bees of the genus *Lithurgus* have not been recorded in Poland. Five females and one male of *L.cornutus* were caught in Lublin Region, SE Poland. The localities are beyond the range of this species, being the northernmost known records from Central Europe. The following information is provided: short diagnosis, ecology, distribution, recent records and threat status of *L.cornutus* in Central Europe.

## Introduction

The genus *Lithurgus* Berthold, 1827 includes 33 species (Gonzalez et al. 2013) distributed in tropical (with exceptions in the wet tropics of the Americas) and warm to moderate temperate zones (Michener 2007). Three species of the genus *Lithurgus: L.chrysurus* Fonscolombe, 1834; *L.cornutus* (Fabricius, 1787) and *L.tibialis* Morawitz, 1875 - were reported in Europe (Michez et al. 2019). Two of them, *L.cornutus* and *L.chrysurus*, occur in Central Europe (Westrich 1990). Until recently, bees from the genus *Lithurgus* have not been recorded in Poland (Borański et al. 2019). However, new localities of *L.cornutus* in Puławy and Lublin, SE Poland have now been discovered. This paper presents the diagnosis, data on biology and general distribution of the species.

## Material and methods

**Figure 1.**
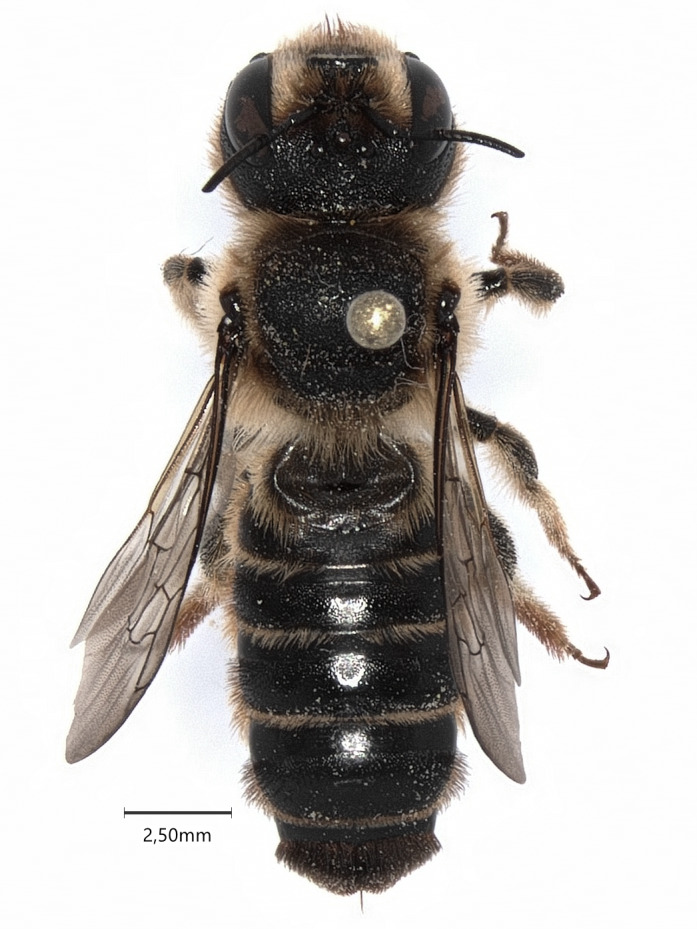
Female of *Lithurguscornutus* (caught in Puławy - 21 July 2021).

**Figure 2.**
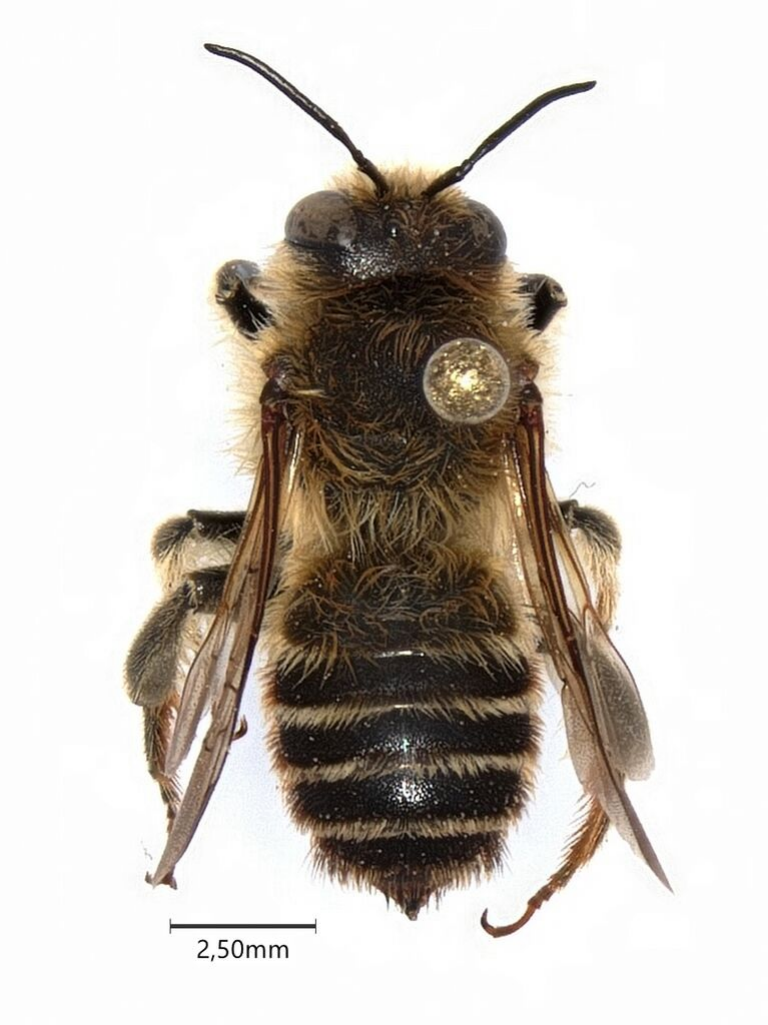
Male of *Lithurguscornutus* (caught in Lublin - 22 July 2021).

During the study on bee diversity conducted in 2020-2021 in SE Poland, five females and one male of *Lithurguscornutus* were collected. The bees were caught with the use of a sweeping net on *Centaureastoebe* Tausch (in Puławy (51.403094N, 21.987693E), 28 July 2020 and 21 July 2021) and *Cirsiumdecussatum* Janka (in Lublin (51.262246N, 22.514108E) - Botanical Garden UMCS 22 July 2021). The species and subspecies of the specimens were identified following van der Zanden (1986) and Scheuchl (2006). The morphometric measurements and photographs were conducted using KEYENCE VHX-970F microscope under 10x magnification. The specimens are housed in the collection of M. Borański (Figs 1, 2). The spectrum of plant species visited by the caught specimens were examined by means of palynological analysis. Pollen loads were collected from the scopa and dusted on to microscope slides. Pollen samples were subsequently protected with a cover slip and glycerol gelatine as a mounting medium (Splitt et al. 2021). For each microscopic slide, at least 300 consecutive pollen grains were checked. Reference slides from our own collection, including pollen samples from native and alien plant species collected in 2015-2021 across rural and urban environments in Poland, as well as detailed morphological data on pollen grains from PalDat (2021), were used to confirm the observations. The IUCN categories of threat are given after Nieto et al. (2014). Status of threat in particular European countries is given after available regional Red Lists or Red Books of threatened species.

## Results and discussion

Lithurguscornutus(Fabricius, 1787)ssp.fuscipennis

**Diagnosis**

Bees of the genus *Lithurgus* can be easily separated from other Central European megachilids by the combination of the following features: lack of arolium, mandibles wider at the base than at the end edge, pygidial plate present, in female facial process well developed (Banaszak and Romasenko 1998, Scheuchl 2006). The key for the Central European species of the *Lithurgus* Berthold is given in Table 1.

The body size of the specimens was highly variable (Table 2). Moreover, especially for the female number 3 and the male, the body length was below the values given for this species. This might be misleading when identifying the specimens found outside of the usual range of occurrence. Therefore, we suggest that the range of the body length in keys should be revised.

Reductions in body size in *L.cornutus* may be a response to environmental and nutritional stresses, which are common in solitary bees (Chole et al. 2019). Although the species seems to tolerate lower temperatures than within the typical range of occurrence, this stressor may negatively affect larval development. Additionally, Puławy and Lublin, the localities where *L.cornutus* specimens were caught, are within one of the most apiary-dense region of Europe (Bieńkowska et al. 2020). Therefore, we assume that *L.cornutus* is under a strong pressure from managed honey bees due to competition over food resources, which also has a negative effect on body size in its offspring.

**Bionomics**

Univoltine. Flight season from July to August (Gogala 2014). The females visit species of Asteraceae, mainly of the subfamily Carduoideae (*Carduus*, *Cirsium*, *Centaurea*, *Onopordum*) (Banaszak and Romasenko 1998, Scheuchl and Willner 2016). However, palynological analysis of the pollen from scopa showed that females of *L.cornutus* gather pollen also from other Asteraceae (*Cichorium*, *Helianthus*, *Taraxacum*), Fabaceae (*Onobrychis*), Chenopodiaceae (*Chenopodium*) and Boraginaceae (*Echium*) (Güler and Sorkun 2007). The bees from Poland were caught while gathering pollen from *Centaureastoebe* (Puławy) and *Cirsiumdecussatum* (Lublin) and palynological analysis of pollen from scopa showed no presence of other species. Most often, *L.cornutus* is referenced as oligolectic (Banaszak and Romasenko 1998, Patchinger 2004, Ban-Calefariu 2009, Gogala 2014, Scheuchl and Willner 2016); however, according to Michez et al. (2019), the species is polylectic. On the other hand, the spectrum of visited plants allows us to classify *L.cornutus* as mesolectic (Cane and Sipes 2006).

*Lithurguscornutus* prefers moist and warm environments especially forest edges (Patchinger 2004). Specimens from Poland were also caught in similar habitats - Puławy - the edge of the mixed forest; Lublin - Botanical Garden. The bees nest in self-created tunnels in dead wood or soft rotten wood (Banaszak and Romasenko 1998). Nests might be parasitised by *Stelissimillima* Morawitz, 1876 (Kasparek 2015). Some authors also report *Stelispunctulatissima* (Kirby, 1802) as a cuckoo bee of *L.cornutus* (Banaszak and Romasenko 1998, Ban-Calefariu 2009, Macek et al. 2010).

**Distribution**

The distribution of *Lithurguscornutus* is centred around the Mediterranean Basin (Michez et al. 2019). This species was reported in North Africa from Morocco and Algeria; in Eurasia, from Portugal through southern and southern-central Europe, Asia Minor, the Caucasus and Central Asia to the mountainous region of Central Siberia (Krasnoyarsk). In Europe, the northern border range of this bee is eastern Austria, Moravia and northern Romania (Scheuchl and Willner 2016). The records of the new localites of *L.cornutus* in Poland, as well as the recent finding from Belarus – Krichev (VE15) (Khvir 2019), suggest the expansion of its range of occurrence to the north, which in turn, may be related to global warming in this part of Europe. Climate change is regarded as one of the major factors triggering the spread of species in new areas and dynamic changes in species distribution in relation to climate warming have already been documented in other bee species (Banaszak et al. 2019, Borański et al. 2019a, Biella et al. 2020).

Analysing the localisation and dispersion of *L.cornutus* in France, Dufrêne et al. (2016) noted that its distribution very often follows the course of rivers. It is in line with the here-presented records of *L.cornutus* in Poland - Puławy (EB69) - Vistula River; Lublin (FB08) - Bystrzyca River, Czechówka River and other recent ones from Central Europe near river valleys: Austria - Vienna (XP04 and XP13) – Danube River (Patchinger 2003, Zettel et al. 2016); Czech Republic - Sedlec (XQ20) – Thaya River (Přidal 2014); Germany - Lampertheim (MV59) – Rhine River (Reder 2020). These data suggest that the species seems to migrate mainly along valleys of rivers. *Lithurguscornutus* is listed on the European Red List of Bees as least concern (Nieto et al. 2014). The species occupies a large range and can be locally abundant (Ortiz-Sánchez and Ornosa 2014), but in southern-central Europe, it occurs very sparsely (Přidal 2014). This species is included in the national Red Lists of the following European countries: Slovenia (vulnerable: Minister za okolje, prostor in energijo 2002), Slovakia (endangered: Lukáš 2001) and the Czech Republic (critically endangered; Straka and Bogusch 2017).

Our paper presents northernmost known records of *L.cornutus* from Central Europe. Although our own data, as well as the reviewed literature, should be treated as predictors of future trends in dispersal of the species, more up-to-date research in Central Europe is needed on the species distribution and migration, as well as on its endangerment status. Thorough studies are required to help guide conservation approaches for *L.cornutus*.

## Figures and Tables

**Table 1.** Key to the Central European species of the *Lithurgus* Berthold.

1	Antennae 12-segmented and metasomal scopa present (females)	2
-	Antennae 13-segmented and metasomal scopa absent (males)	3
2	Facial process small, its front edge flat, upper edge rounded. Clypeus densely and strongly punctate. 6^th^ tergite of metasoma with dense reddish hairs. Smaller species: 13-15 mm	*L.chrysurus* Fonscolombe, 1834
-	Facial process large its front edge concave, upper edge slightly indented. Clypeus almost not punctate. 6^th^ tergite of metasoma with dark brown hairs. Larger species: 16-20 mm	*L.cornutus* (Fabricius, 1787)
3	Smaller species: 11-13 mm; basal part of metasomal terga 2-4 with white or yellow hairs	*L.chrysurus* Fonscolombe, 1834
-	Larger species: 14-15 mm; basal part of metasomal terga 2-4 with darker hairs (brown or reddish)	*L.cornutus* (Fabricius, 1787)

**Table 2.** Metrical characters of caught bees of Lithurguscornutusssp.fuscipennis.

Character	x (in millimetres)						
	1	2	3	4	5	mean ± SD	6
Length of body	18.67	19.3	13.91	15.92	16.32	16.82 ± 1.95	12.6
Length of head	4.89	4.55	3.49	3.99	4.0	4.18 ± 0.49	3.1
Width of head	5.57	5.46	4.5	4.8	4.69	5.00 ± 0.43	3.79
Length of metasoma	10.51	10.74	6.5	7.91	8.18	8.77 ± 1.62	6.62
Width of metasoma	5.79	5.25	4.66	5.09	5.25	5.21 ± 0.36	4.42

1. Female caught in Puławy 2020; 2. Female caught in Puławy 2021; 3-5. Females caught in Lublin 2021; 6. Male caught in Lublin 2021
